# Monthly Summary

**Published:** 1868-09

**Authors:** 


### MONTHLY SUMMARY.

DR. BALLARD's nine aphorisms on the influence of weather upon sickness are thus given in his recent Report on the Health of Islington for 1867; "1. That an increase of atmospheric temperature is normally associated with an increase of general sickness. 2. That a decrease of atmospheric temperature is normally associated with a diminution of general sickness. 3. That for the most part the increase or decrease of sickness is proportional in amount to the extent to which the atmospheric temperature rises or falls. 4. That it is an error to suppose (as is populaly held) that sudden changes in temperature are (as a rule) damaging to public health. A sudden change from cold to hot weather is indeed very damaging; but a sudden change from hot to cold is one of the most favorable circumstances that can occur when sickness is regarded broadly as respects a large population. 5. That, remarkably enough. these influences are most marked in the directions I have mentioned in the colder season of the year, and more certain in the winter than in the summer. 6, That rises and falls of temperature are more certain and effectual in their special operation upon public health when at the same time the daily range of temperature is lessened, than they are when the daily range is at the same time increased; rises of temperature increasing sickness more certainly and markedly, and falls of temperature decreasing it more certainly and markedly. 7. That a fall of rain lessens sickness generally, sometimes immediately, sometimes after a short interval, and that. as a rule, the reduction of general sickness is greater when the fall of rain is heavy than when it is light. 8. That drought, on the other hand, tends to augment general sickness. 9. That wet weather in the summer season operates more certainly in improving public health than it does in the winter season."—*Med. Times and Gazette.*

*Cystic Tumor of the Lower Jaw. Removal of the arger Part of the body of the Bone*—J. F., æt. 18 from Batesville, Miss., entered Prof. Stone's wards in the Charity Hospital, December 7, 1867, for tumor of the lower jaw. The disease was ascribed to a blow received upon the chin two yerrs previously, since which time the bone had been steadily enlarging. Upon examination by Prof. Stone and myself, the enlargement was found to involve both the alveolar and basilar divisions of the bone, from the symphisis to within a short distance of the ramus upon the right, and to the first large molar tooth upon the left side. The tumor thus formed did not xeceed the size of a pullet's egg, but projected almost altogether towards the cavity of the mouth, where it interfered very uncomfortably with mastication and the movements of the tongue. It had never been the seat of any considerable pain, and there was no evidence whatever of contamination of the cervical lymphatic glands or of any constitutional cachexy. Under firm pressure it was ascertained to consist of a thin shell of bone enclosing a semi-solid elastic tissue.

Taking thus into consideration the situation of the growth, its painless character, its comparatively slow increase, its semi-fluid consistence, the absence of any local or constitutional contamination, and its indisposition to ulcerate, the disease was unhesitatingly pronounced cystic tumor of the bone, most probably of the myeloid variety. Removal by the knife was therefore advised, with the assurance that a return would not be likely to occur, as such tumors seem to be generally benign.

By the request of Prof. Stone, I performed the operatation, in the presence of the medical class of the University, with the assitance of Dr. Warren Stone, Jr., and others, on the 9th of December. The method' pursued after placing the patient under the influence of chloroform, consisted in dividing the skin by a single curved incision along the base of the jaw, extending nearly to the ramus on each side; dissecting back the soft parts in front and below; and then dividing the body of the bone. by means of a small saw, a short distance beyond the limits of the enlargement. This having been done. and a ligature passed through the tongue and confided to an assistant to prevent retraction of the organ and consequent suffocation. firm traction was made upon the mass at one end, and its separation rapidly effected by dividing the mylo-hyoid, genio-hyoid and genio-glossal muscles close to their bony attachments.

The operation was accompanied and followed by a frightful hæmorrhage, the blood welling up from numberless vessels too small to be secured by ligatures; but by a free use of iron alum and filling the gaping wound with broken ice the flow was gradually arrested, but not until the patient was apparently almost exsanguious. It was now also found that one of the sawn ends of the bone presented an unhealthy appearance, and the further removal of half an inch became necessary; but this was readily effected by the use of cutting forceps. After reaction had been established by means of frictions, hot bricks and stimulants, the edges of the wound were brought together with twisted sutures, and the point of the tongue confined outside of tne mouth by attaching the ligature to a strip of adhesive plaster placed upon the chest. This latter precaution was absolutely necessary, for it was found by experiment that as soon as the traction was relaxed the organ was drawn back into the throat and threatened suffocation. By careful attention upon the part of Mr. Watkins, the student of the ward, the case did well, and at the end of three days, the patient beimg able to control his tongue, the ligature was removed. The sutures were removed upon the fourth and fifth davs, entire union having taken place, and in twelve days from the time of the operation the patient returned home.

Examination of the tumor subsequent to its removal proved the correctness of the diagnosis, for notwithstanding the close resemblance of the contents of the bone cyst to the brain-like substance of medullary cancer, the microscopic elements were those of myeloid tumor. In farther proof of its benign nature, it may be stated that it is now six months since the operation, and there is not the slightest evidence of any dispesition to a recurrence which would scarcely be the case in encephaloid.—*New Orleans Jour. of Medicine*

---

An electrical probe has been devised in France for the examination of gun-shot wounds. When the points of the instrument come in contact with a metallic body, a circuit is made, and the fact announced by the ringing of a small bell.—*Med. Gazette.*

*Tumors of the Tongue and Pharynx.*—The Paris correspondent of the *Medical Record* communicates the following:

" M. Desgranges publishes in the *Journal de Lyon*, certain considerations on tumors of the tongue and pharynx, and a special method for operating upon them. This method belongs to M. Sedillot, and consists of a section of the lower maxilla on the median line, by means of which the two halves of the bone could be drawn aside and sufficient space left to excise the tumor. The wound of the soft parts heals readily, but for the cicatrization of the segments of the maxilla it was found necessary to maintain the adjustment by means of pincers. This instrument presents certain inconveniences, and M. Desgranges has used metallic sutures instead, piercing the bone with a drill, for the passage of the silver wire.

" Two cases are related where this operation was successfully performed for an epithelial cancer of the floor of the mouth. In the first case, the tumor, situated under the tongue, extended form the first molar of the left side to the canine at the right. The posterior face of the maxilla was invaded, and the incisors and left canine were partially loosened from the alveoli.

" In operating, the integument was divided as far as the hyoid bone, then the section of the maxilla affected by the chain saw. Care was taken that the section should be made at the left side, and the insertions of the genio-hyoid and genio-glossal muscles of the right side avoided. Upon separating the segments of the bone, the diseased parts were easily removed with curved scissors, without touching the subjacent muscles. No blood fell into the pharynx, so that suffocation was avoided. The results were most happy. The tongue retained its movements, and no trouble occurred in the respiration. The two halves of the maxilla were not displaced, and when the patient left the hospital three weeks after the operation, a fibrous callus united the segments, and with sufficient solidity to permit movements of the entire jaw.

" In the second case, the tumor had burrowed more deeply, and was ulcerated. The superficial layers of muscles were removed but enough remained to insure the movements of the tongue. The operation, performed exactlv as in the preceding case, was followed by a slight attack of erysipelas, and it was a month before the two halves of the divided maxilla ceased to shake in the movements of the lower jaw. But in six weeks the osseous union was complete.

"This preliminary osteotomy opens a free route to the bistoury ; it enables the operator to examine tbe entire tumor, and to persue its prolongations, a circumstance essential as a guarantee against relapse. Moreover, the extreme difficulty of ligating numerous arteries encountered in this region is greatly palliated and finally the danger avoided of suffocation during the anæsthetic sleep, on account of blood flowing into the larynx."—*Med. Gazette.*

---

*Simple Synthesis of Urea.*—Professor Kolbe, of Leipzig, who is at present in London, described at the last meeting of the Chemical society an interesting and wonderfully simple process for the synthesis urea. All chemists know that molecules of carbonate of ammonia contains the elements of a molecule of urea *plus* two molecules of water. During the incipient putrefaction of urine, urea actually takes up two molecules of water, according to the well-known formula

$$COH_4 N_2 + 2H_2 O = (NH_4)_2 CO_3 ,$$

and is thus converted into carbonate of ammonia. The same change can be effected by heating an aqueous solution of urea to a temperature of 284° Fahr. in a hermetically sealed tube.

Urea, in fact, bear to the carbonate of ammonia the same relation that oxamide does to oxalate of ammonia. Now when oxalate of ammonia is heated, it loses two molecules of water, and is converted into oxamide ; but carbonate ammonia, when exposed to similar treatment, is simply volatilized instead of being dehydrated. The parallel reaction has, however, at last been effected by Professor Kolbe, and thus this important gap in the history of urea is filled up. The process is very simple. Dry carbonate of ammonia is inclosed in a hermetically sealed glass tube, and heated for some time to a temperature short of that at which urea decomposes. Under these circumstances, the salt behaves like oxalate of ammonia—that is to say, it loses water and becomes converted into corresponding amide—in other words, into urea. The formula is, of course, precisely the reverse of that given above, but we will vary its form by presenting it in the old notation, which is, even now, more familiar to many of our readers—

$$2(NH_4 O, CO_2) = C_2 O_2 H_4 N_2 + 2H O_2 .$$

As usual, the thing being done, one is tempted to wonder why so very simple an experiment was never tried before.—*Med. Times and Gaz.* April 18, 1868.

*The Foot.*—It may be doubted whether there exists throughout the whole civilized world a well formed foot. Many exquisites of both sexes claim admiration for their pedal extremities, but it is the boots and shoes which cover them which we are called on to admire. Their feet, if bared would present a very great divergence from the classical idea of beauty. The firmly-planted foot, neither too large nor too small, but justly proportioned to the height and weight it sustains, the smooth surface and regular curved lines, the distinctness of the divisions and the perfect formation of each toe, with its well marked separativeness, and its gradation of size and regularity of detail, to the very tip of the nail, are now to be seen only in art. In Greek nature they were found, for the ancient sandal, which left the foot unfettered, gave freedom to the development of its natural grace and proportions. The modern boot or shoe, with the prevalent notion that every thing must be sacrificed to smallness, has squeezed the foot into a lump as knotty and irregular as a bit of pudding stone, where the distorted toes are so imbedded in the mass and mutilated by the pressure, that it is impossible to pick them out in the individuality and completeness of their original forms.

As our coarse climate forbids the sandal, and renders the shoe necessary, care should be taken to adapt it as perfectly as possible to the natural conformation of the foot. It should be long and wide enough to admit of a free play of the toes; the space between the heel and sole of the shoe should be firm and of a curve the same height as the natural foot, while no part of the artificial covering should be so binding as to prevent the free action of the muscles and circulation of the blood.

---

*Compound Fracture of the Lower Jaw.*—Mary R., a stout Irishwoman, applied as an out-door patient at hospital, March 1, for injuries about the face, produced by falling head foremost down stairs upon a brick pavement. Examination showed complete division of the upper lip into the left nostril, and oblique fracture of the inferior maxilla upon the corresponding side of the symphisis. The fragments of the broken jaw were not in apposition but overlapping, one of them having been evidently driven in by the fall, producing great laceration of the gums. With some effort the broken ends were reduced and brought into contact; but owing to the obliquity of the line of fracture and the contraction of the muscles attached to the longer fragment, displacement recurred so soon as the hands were removed. Several and various attempts to keep the parts in proper position having failed, it was determined to stitch the wound in the upper lip, and after this was healed to have an interdental splint applied to the fractured bone, the parts being in the meantime gently supported by a compress beneath the chin, confined by a bandage over the head. The lip having completely united in the course of four or five days, the patient was taken to Dr. J. E. Kells, dentist, who took the necessary casts and constructed npon them an interdental splint of vulcanized rubber. By this means the fragments were brought into perfect apposition, and so maintained for three or four weeks, at the end of which time complete union had taken place.

The interdental splint, it may not be generally known, was invented by Dr. J. B. Bean, dental surgeon in the Confederate States Army, and is one of the most valuable additions made to the surgical *armamentarium* during the past ten years. A detailed account of its operation may be found in the New Orleans Medical and Surgical Journal for November, 1866, by Dr. Warren Stone, Jr. I take the liberty of also stating that Dr. Kells will cheerfully demonstrate the process to any one who may call upon him.—*N. Orleans Jour. of Medicine*

---

*Iodoform*—This substance, in powder, has recently been applied, in Paris, with great success, to the surface of obstinate ulcers. It rapidly causes them to heal. The *Journal of Applied Chemistry* remarks of it: "In addition to the virtues which it possesses in common with iodine, it is very useful as an anodyne, and especially in neuralgic affections. In many local diseases it is employed with good success. It possesses anæsthetic properties when volatilized and inhaled, though to a degree inferior to chloroform or ether. On the score of economy, it is fully equal to iodine or any other of its compounds, and far preferable to most of them, on accont of being a non-irritant, and becoming more readily absorbed and assimilated in the system. We trust that its use will become more extensive, and would urge physicians to test its qualities more thoroughly."

*Insalubrity of Cast Iron Stoves.*—" When the attention of the Academy of Sciences of Paris was drawn some time since by M. Carret, one of the physicians of the Hotel Dieu of Chambery, in several papers, to the possible evil consequences of the use of cast iron stoves, but little interest was excited in the matter. Recently General Morin has again brought the subject forward with better success. M. Carret does not hesitate to assert most positively that cast-iron stoves are sources of danger to those who habitually employ them. During an epidemic which recently prevailed in Savoy, but upon which M. Carret does not furnish us with any detailed information, he observes that all the inhabitants who were affected with it made use of cast-iron stoves, which had lately been imported into the country, whereas all those who employed other modes of firing, or other sorts of stoves, were left untouched by the disease. An epidemic of typhoid fever, which broke out some time after at the Lyceum of Chambery, was regarded by the same author as being influenced by a large cast-iron stove in the children's dormitory. General Morin speaks in the highest terms of M. Carret's memoirs, to which the recent experiments of MM. Trorst and Deville give additional importance. These able investigators have established that iron and cast-iron when heated to a certain degree becomes pervious to the passage of gas. They have been enabled to state the quantity of oxide of carbon which may, as they suppose, transude from a given surface of metal, and have shown that the air which surrounds a stove of cast-iron is saturated with hydrogen and oxide of carbon. They conclude that cast iron stoves when sufficiently heated absorb oxygen, and give issue to carbonic acid. General Morin related some comparative experiments which had been performed by M. Carret, and which he said, corroborate this theory. Thus, after having remained during one full hour in a room heated to 40° (centigrade) by means of a sheet iron stove, M. Carret perspired abundantly, got a good appetite, but felt no sickness whatever ; he had obtained the same result with an earthenware stove ; but the experiment, when performed during only one half hour with a cast-iron stove, had brought on intense headache and sickness. M. Deville, at the same sitting of the Academy, supported these views with considerable warmth. The danger which attended the use of cast-iron stoves, he said, was enormous and truly formidable. In his lecture-room at the Sorbonne he had placed two electric bells, which were set in motion as soon hydrogen or oxide of carbon was diffused in the room. Well, during his last lecture the two cast-iron stoves had scarcely been lit when the bells began to ring.

"These facts are certainly startling, if we consider the reputation of comparative harmlessness which these articles of domestic use had hitherto enjoyed. In France, particularly, the lodgings of the poorer classes, the barrack-rooms of the soldiery, the artist's studios, the class-rooms of large schools, &c., are commonly heated by this means. Of course, we are inclined to question M. Carret's conclusions; but the apprarently accurate character of the facts recorded, joined to the authority of those who have brought them forward, demand for them a serious investigation. We are glad to be able to add that a committe has been appointed by the Academy for the purpose of examining thoroughly into the subject. This committee is composed of MM. Claude Bernard, Morin, Fremy, Deville, and Bussy, and we shall not fail, when the time comes, to mention what shall have been the result of their researches."—*Lancet.*

---

*Hereditary Nature of Hare-lip.*—M. Demarquay lately asked the advice of the members of the Surgical Society of Paris touching a little girl, five years old, who presented a double hare-lip. Some difficulties will be encountered in the operation, but the interest of the case lies in the fact that, in the family from the grand-parents downwards, eleven children have been born with hare-lip, or with a peculiar conformation of the lower lip—namely, two openings on either side of the mesial lines traversing the whole labial thickness, with a peculiar form of the lip itself. To this latter defect M. Demarquay had called attention, in the *Gazette Medicale*, as early as 1845.—*Lancet.*

---

*Premature Interment.*—All risk of premature interment may be obviated, according to Dr. Martenot, chief physician of the Hospital de Lyons by the following test; Produce a blister on one of the fingers or toes by holding a flame of a candle in contact with it for some seconds. If the vesicle thus formed contain serous fluid, life still exists. If it be merely filled wit vapour we may be sure of death. This sign is said to be unering. "A dry vesicle, death; a liquid vesicle, life."—*Med. Gazette.*

